# The East Bay Diesel Exposure Project: a biomonitoring study of parents and their children in heavily impacted communities

**DOI:** 10.1038/s41370-023-00622-1

**Published:** 2023-12-15

**Authors:** Daniel Sultana, Duyen Kauffman, Rosemary Castorina, Michael H. Paulsen, Russell Bartlett, Kelsey Ranjbar, Robert B. Gunier, Victor Aguirre, Marina Rowen, Natalia Garban, Josephine DeGuzman, Jianwen She, Regan Patterson, Christopher D. Simpson, Asa Bradman, Sara Hoover

**Affiliations:** 1 Office of Environmental Health Hazard Assessment (OEHHA), California Environmental Protection Agency, Oakland, CA USA; 2 Environmental Health Investigations Branch, California Department of Public Health, Richmond, CA USA; 3 Center for Environmental Research and Community Health (CERCH), School of Public Health, University of California, Berkeley, CA USA; 4 Department of Environmental and Occupational Health Sciences, School of Public Health, University of Washington, Seattle, WA USA; 5 Environmental Health Laboratory Branch, California Department of Public Health, Richmond, CA USA; 6 Department of Civil and Environmental Engineering, University of California, Berkeley, CA USA; 7 Department of Public Health, University of California, Merced, CA USA

**Keywords:** Biomonitoring, Human exposure, Diesel exhaust, 1-nitropyrene, Urinary metabolites, Children

## Abstract

**Background:**

Diesel exhaust (DE) exposures pose concerns for serious health effects, including asthma and lung cancer, in California communities burdened by multiple stressors.

**Objective:**

To evaluate DE exposures in disproportionately impacted communities using biomonitoring and compare results for adults and children within and between families.

**Methods:**

We recruited 40 families in the San Francisco East Bay area. Two metabolites of 1-nitropyrene (1-NP), a marker for DE exposures, were measured in urine samples from parent–child pairs. For 25 families, we collected single-day spot urine samples during two sampling rounds separated by an average of four months. For the 15 other families, we collected daily spot urine samples over four consecutive days during the two sampling rounds. We also measured 1-NP in household dust and indoor air. Associations between urinary metabolite levels and participant demographics, season, and 1-NP levels in dust and air were evaluated.

**Results:**

At least one 1-NP metabolite was present in 96.6% of the urine samples. Detection frequencies for 1-NP in dust and indoor air were 97% and 74%, respectively. Results from random effect models indicated that levels of the 1-NP metabolite 6-hydroxy-1-nitropyrene (6-OHNP) were significantly higher in parents compared with their children (*p*-value = 0.005). Urinary 1-NP metabolite levels were generally higher during the fall and winter months. Within-subject variability was higher than between-subject variability (~60% of total variance versus ~40%, respectively), indicating high short-term temporal variability.

**Impact:**

Biomonitoring, coupled with air monitoring, improves understanding of hyperlocal air pollution impacts. Results from these studies will inform the design of effective exposure mitigation strategies in disproportionately affected communities.

## Introduction

Diesel exhaust (DE) exposures vary widely in California, with low-income communities and communities of color often experiencing disproportionately higher exposures [1, 2]. The harmful effects of DE, including asthma, cancer, and cardiovascular disease [3–7], can be exacerbated by the multiple environmental, health, and social stressors faced by these communities [8]. Although regulations in California (13 CCR § 2025) have reduced emissions from diesel-powered vehicles overall, recent studies have found that heavily impacted areas, such as West Oakland, experience highly elevated air pollution in neighborhoods near traffic, rail, and maritime sources [9–11].

“Diesel exhaust” (i.e., the entire complex mixture) was recommended in 2009 as a priority chemical for the California Environmental Contaminant Biomonitoring Program (CECBP or Biomonitoring California) by the program’s Scientific Guidance Panel (SGP), a legislatively mandated collaboration between OEHHA, the Department of Toxic Substances Control, and the California Department of Public Health (CDPH). The SGP reviews evidence for the degree of exposure, toxicity, and the ability to detect biomarkers at levels relevant to the general population when considering chemicals for the priority list.

Based on reviews of the scientific literature [4, 12–17] and a series of discussions with invited experts at SGP meetings from 2008 to 2016, metabolites of 1-nitropyrene (1-NP) were identified as the most viable biomarkers for DE exposures. 1-NP is preferentially formed by high-temperature combustion processes in diesel engines and is the most abundant nitro-polyaromatic hydrocarbon (PAH) observed in airborne particulate matter (PM) from DE [18, 19]. Diesel emissions are the major source of 1-NP [20, 21]. 6-Hydroxy-1-nitropyrene (6-OHNP) and 8-hydroxy-1-nitropyrene (8-OHNP) are commonly measured urinary metabolites of 1-NP [13, 17, 22]. These are useful biomarkers because they reflect personal DE exposures, and urine samples are relatively easy to collect.

Positive associations between urinary 1-NP metabolites and airborne 1-NP were found in individuals who commute across the U.S.–Mexico border at San Ysidro, CA [13]. A small pilot study of children in the Salinas Valley compared with children in Oakland found higher levels of 6-OHNP and 8-OHNP in the Oakland population [23], as expected based on greater diesel vehicle traffic in the urban area. That pilot study informed the design of the current East Bay Diesel Exposure Project (EBDEP), which was launched in 2017.

This is the first study to evaluate DE exposures in families with young children by pairing 1-NP biomonitoring with measurements in household dust and indoor air. We report results for 6-OHNP and 8-OHNP in urine samples from 40 parent–child pairs residing in Oakland, Richmond, and other nearby communities in the San Francisco East Bay. We also describe short-term within- and between-subject variability of the metabolite levels and summarize measurements of 1-NP in dust and indoor air from the same households. The ultimate goal of this work is to support efforts to reduce DE exposures in communities heavily burdened by air pollution and other health, environmental, and social stressors.

## Materials and methods

### Study participants and recruitment

We used the diesel PM indicator from CalEnviroScreen 3.0 [24] to identify geographic areas in the San Francisco East Bay with a range of potential DE exposures. CalEnviroScreen is a mapping tool developed by OEHHA to help identify California communities heavily impacted by multiple pollution sources and other stressors. The CalEnviroScreen 3.0 diesel indicator represents modeled diesel PM emissions from 2012 for California census tracts. We primarily targeted recruitment in areas with higher estimated DE exposures, such as neighborhoods near the Port of Oakland and interstate highways and included several nearby areas with lower estimated exposures for comparison (see Figs. S1 and S2 in the Supplementary Material [SM]).

Outreach was conducted primarily in person at community organizations, health clinics and family practices, local libraries, and childcare centers. We also posted study flyers in these settings and staffed informational tables at these and other public locations and events to recruit potential participants.

Forty parent–child pairs (*n* = 80 total) participated in the study. All participants spoke English, had toilet-trained children aged 2–10 years old, and were intending to remain at their current home for at least four months after the first sampling round. Both males and females were enrolled.

Sampling occurred between January 2018 and February 2019. The study was originally designed to compare DE exposures during seasons with higher air pollution (generally fall and winter) with exposures during lower pollution seasons (generally spring and summer), but this was not implemented due to slower than anticipated recruitment and challenges with scheduling home visits.

All study instruments and activities were approved by the UC Berkeley and California Health and Human Services Agency Institutional Review Boards (IRBs) that oversaw EBDEP. Parents provided written informed consent and children 7–10 years old provided assent to participate.

As mandated by the program’s enabling legislation, the consent form included the option for parents to receive their and/or their child’s individual biomonitoring results. Parents could also choose to donate their family’s urine samples for future chemical analyses.

### Sampling rounds

Each family participated in two four-day sampling rounds separated by an average of four months (range: 0.5–8 months). SM Table S1 summarizes the timing of sample and data collection. On day one of each family’s first sampling round, study staff enrolled and obtained consent from the parent; measured the height and weight of the child and parent; administered the initial exposure questionnaire to the parent; provided GPS location loggers (one for the parent and one for the child) to be carried during the sampling round; instructed the parent on how to complete daily time-activity diaries for themselves and their child; completed a home walk-through; deployed air monitoring equipment; and collected a vacuum bag or dust sample from bagless vacuums when available. On day four of the first sampling round, a follow-up exposure questionnaire was administered to assess potential DE exposures that may have occurred after day one; staff also collected urine samples from the child and parent, floor sweepings from households that did not have a vacuum, and the air monitor.

For the second sampling round, staff followed the methods described above, except dust sampling was not repeated.

### Exposure questionnaire and home walk-through

The exposure questionnaires were administered during both sampling rounds to collect information about demographics and potential determinants of 1-NP exposures, including parent occupation and use of diesel-powered equipment; parent’s work location(s) and the child’s school or childcare location; time spent at work, school, or childcare (to supplement information collected via the GPS loggers and time-activity diaries); smoking exposures (active or secondhand); sources of combustion byproducts in or around the home, such as a gas appliance, fireplace, or grill; type of vehicle; whether there was an attached garage; use of a stove fan; and other housing characteristics. Home walk-throughs were conducted on day one of each sampling round to collect information potentially relevant to 1-NP exposures, such as type of residence, type of heating system, presence of a stove fan and whether it vents to the outside, and use of portable air cleaner(s).

### Sample collection and analysis

#### Urine

On day one of each sampling round, parents were trained on urine collection and provided with sampling supplies. For 25 of the families, parents collected one or more spot urine samples from themselves and their children on day four of each sampling round. The 15 other families collected one or more spot samples (usually a first morning void) every day for four days during each sampling round. Urine samples were refrigerated after collection, retrieved by study staff on day four of each sampling round, and transported in a cooler on frozen gel packs to the Environmental Health Laboratory (EHL) at CDPH. EHL aliquoted the samples, measured specific gravity using a refractometer, measured creatinine using a colorimetric assay based on the Jaffe reaction, and stored the aliquots at −80 °C.

Aliquots (of 100 ml if possible; less if only low-volume samples were available) and the field blanks were shipped on dry ice to Dr. Christopher Simpson’s laboratory at the University of Washington (UW) for 1-NP metabolite analyses. Samples were stored at −20°C until preparation for analysis. Two samples were lost at UW due to vial breakage. The 6-OHNP and 8-OHNP analyses were performed using high-performance liquid chromatography with tandem mass spectrometry (HPLC-MS/MS). Preparation steps included enzymatic hydrolysis, solid phase extraction using blue rayon, and cleanup with alumina [15, 17].

Three hundred thirty-five urine samples from the 40 families and 19 field blanks were analyzed by UW. Six of these samples were formed by combining two low-volume samples collected from a single participant on the same sampling day. Forty-two 6-OHNP and 17 8-OHNP measurements were excluded as not valid, resulting in 155 and 168 valid measurements for 6-OHNP and 8-OHNP in children, and 138 and 150 valid measurements for 6-OHNP and 8-OHNP in parents. There were 138 valid parent–child paired measurements for 6-OHNP and 164 valid parent–child paired measurements for 8-OHNP. The reasons for excluding some results as not valid included chromatographic interferences and poor recovery of internal standards.

Quality control (QC) samples included field blanks (laboratory-grade deionized water), internal standard-spiked laboratory water blanks, benchmark urine, and analyte-spiked benchmark urine. The benchmark urine was a mixture of residual de-identified samples from prior studies. This use of the samples was in compliance with IRB approvals for those studies.

The limits of detection (LODs) were based on sample volumes of 100 ml and were defined as the average plus one standard deviation (SD) of the internal standard-spiked laboratory water blanks. The LODs for 6-OHNP and 8-OHNP were 15.5 pg/L and 21.2 pg/L, respectively. Average recoveries for analyte-spiked samples were 126% and 142% for 6-OHNP and 8-OHNP, respectively. Metabolite concentrations in 19 field blanks randomly analyzed along with the participants’ urine samples were all below the LODs. A detailed description of laboratory methods can be found in Toriba et al. [17].

Metabolite concentrations were adjusted using specific gravity (SG) to account for urine dilution according to the equation shown below [25]. We used SG instead of creatinine to adjust concentrations for comparisons between adults and children because age has a significant impact on urinary creatinine levels [26].

$${{{{{{\rm{Concentration}}}}}}}_{{{{{{\rm{SG}}}}}}-{{{{{\rm{adj}}}}}}}={{{{{{\rm{Concentration}}}}}}}_{{{{{{\rm{measured}}}}}}}\times \frac{1.017-1}{{{{{{\rm{SG}}}}}}-1}$$

The median specific gravity for adults (18+; weighted data) of 1.017 in the National Health and Nutrition Examination Survey 2007–2008 cycle [27] was selected as the reference value for the above equation.

#### Household dust

During the first sampling round, a vacuum bag or dust from a bagless vacuum (*n* = 30), or a sample of floor sweepings from households with no vacuum (*n* = 10), was collected from each home and transported on frozen gel packs in a cooler, held in a freezer as needed for interim storage, and delivered to EHL. Dust samples were stored at -20°C at EHL until shipment on frozen gel packs to UW, where they were stored at −20°C until analysis. UW developed methods for measuring 1-NP in dust for this study. Samples were sieved to 150 µm to remove material such as hair, carpet fibers, and large particles [28]. Two hundred mg of sieved dust was then weighed into silanized glass centrifuge tubes (Kimble 73785-5), spiked with deuterated 1-NP internal standard, and extracted by sonicating in 7 ml methylene chloride for 60 min. The extract was then centrifuged for 30 min at 2000 × *g*, and the supernatant removed and concentrated under nitrogen at 45°C in a Turbovap. The concentrated extract was loaded onto silica gel SPE tubes (Supelco, 505048, 3 ml, 500 mg), which had been preconditioned with methylene chloride followed by pentane. Loaded tubes were washed with 1 ml pentane, then eluted with 9 ml of a mixture of methylene chloride and pentane (35:65). The extract was evaporated to dryness under nitrogen at 45°C in a Turbovap and reconstituted in a 1 ml solution of 75:25 ethanol:20 mM sodium acetate at pH 5.5. The final extract was filtered using a 1 ml polypropylene syringe fitted with a 0.2 µm polytetrafluoroethylene (PTFE) filter, and 25 µl of the extract was analyzed by two-dimensional HPLC-MS/MS. Dust concentrations are reported in pg/g, calculated based on the mass of dust.

QC samples included the use of laboratory blanks and spiked benchmark dust samples for repeat analysis. The benchmark dust samples were drawn from a bulk sample provided by study staff. The arithmetic mean spike recovery from silica gel was 105% and the coefficient of variation (CV) was 8%. The CV for replicate analyses of 5 benchmark dust aliquots was also 8%. 1-NP was not detected in the lab blanks, thus the LOD was defined as the lowest calibration standard (15 fg/mg). Interferences and insufficient mass prevented quantification in several dust samples, leaving a total of 36 valid measurements from 33 homes.

#### Indoor air

An Aerosol Black Carbon Detector (ABCD) was placed in participants’ homes to monitor black carbon air concentrations and sample air for measurement of 1-NP [9, 29]. The ABCD was deployed in each family’s home on day one and retrieved on day four of each sampling round. The ABCD computes real-time black carbon concentrations at 5-s intervals based on optical reading of particles collected on a Teflon-coated glass fiber filter (Pall Life Sciences Emfab Filter (99.9% particle retention), Ann Arbor, MI). The ABCD draws air at approximately 111 cc/min for a mean (SD) volume of 0.49 (0.03) m^3^ sampled during each period. After the black carbon data was downloaded, the filters were removed from the ABCD and shipped to the UW laboratory for analysis of 1-NP. Filters were analyzed according to methods described previously [30]. Briefly, preparation involved spiking the filter with a deuterated 1-NP internal standard, sonication with methylene chloride, evaporation, reconstitution in a mixture of ethanol and sodium acetate buffer, filtering of the final extract, and analysis by two-dimensional HPLC-MS/MS.

Stability of the HPLC/MS/MS system was monitored via repeated analysis of a mid-level (50 fg/µL) calibration standard throughout the analytical sequence. QC samples included clean blank filters (“lab blanks”) of the same type used in this study and spiked filters. The average spike recovery from 4 filters was 100% with a CV of 3%. The LOD was defined as the average lab blank plus one standard deviation of the lab blanks. For 1-NP, the LOD was 0.164 pg/filter. Out of a possible 80 filter samples (i.e., one from each household at the end of two sampling rounds), there were 74 valid measurements. Two participants only participated in the first sampling round and an additional 4 measurements were excluded as not valid (due to interference, unknown end time of the sample, or other QC issues). 1-NP air concentrations are reported in pg/m^3^, calculated based on the air volume sampled.

### Data analysis

Statistical analyses were performed using R version 3.6.1 [31] and SAS software version 9.4. Summary statistics were computed for urinary metabolite, dust, and air measurements. Concentrations less than the LOD were imputed as the LOD divided by the square root of two [32]. We calculated weighted Pearson correlation coefficients between log-transformed metabolite levels in parents and children, and separately between log-transformed metabolite levels and the log-transformed air and dust levels of 1-NP. The weights were the inverse of the number of repeated measurements collected from the individual or family.

We used random-effects models and linear mixed-effects models (hereafter referred to as mixed models) to analyze log-transformed metabolite data. The random-effects models included an intercept, a random effect to account for multiple samples from participants, and an error term to account for unexplained variability. The mixed models included a fixed effect for the factor of interest, a random effect to account for multiple samples from participants, and an error term to account for unexplained variability. The error terms were assumed to be uncorrelated.

More specifically, random-effects models were used to estimate the geometric means (GMs) of 6-OHNP and 8-OHNP, and associated 95% confidence intervals (CIs), separately for parents and children. A random-effects model was also used to analyze the differences between metabolite levels in parent–child pairs. Mixed models were used to separately analyze the bivariate associations between urinary 6-OHNP and 8-OHNP levels as dependent variables, with each of the following independent variables: race/ethnicity, family income, and seasonal period. We defined the seasonal periods as fall/winter (1/9/2018–3/19/2018 and 9/22/2018–2/1/2019) and spring/summer (3/20/2018–9/21/2018).

To evaluate the short-term variability of urinary metabolites in the participants who provided daily samples (*n* = 15 parent–child pairs; 215 samples), we calculated the intraclass correlation coefficient (ICC) from the total and within- and between-subject variance using mixed models with a fixed effect for sampling round. The ICC is the ratio of between-subject variance to total variance. An ICC ≥ 0.75 indicates excellent “reproducibility,” which refers to the consistency between the same quantitative measurements repeated multiple times. A higher ICC occurs when within-subject variance is relatively small. An ICC value between 0.4 and 0.75 indicates fair to good reproducibility, and an ICC < 0.4 indicates poor reproducibility [33].

To investigate the relationship between CalEnviroScreen 3.0 diesel PM scores and levels of 1-NP in dust and air and 1-NP metabolites in participants’ urine, we divided participant census tracts into two groups: those with diesel PM scores below the 90th percentile among all California census tracts, and those at or above the 90th percentile. A t-test was used to compare log-transformed dust 1-NP levels in these two groups. A mixed model was used to compare log-transformed indoor air 1-NP levels in the two groups. Mixed models were used to compare log-transformed urinary 1-NP metabolites between these two census tract groups, with and without seasonal effects in the models.

## Results

### Demographic characteristics

Table 1 presents the demographic characteristics of EBDEP participants. Thirty-eight parents identified as female and two as male, with a mean age of 36.6 years. The majority of parents were Hispanic/Latino or White (40% and 35%, respectively), and 20% were Black/African American. Most were highly educated, with 80% completing at least some college. Nearly half worked from home or were stay-at-home parents (47.5%). Children were nearly evenly split between male and female (47.5% and 52.5%), and 82.5% were between 2 and 5 years old (mean [SD] = 4.8 [2.1]; range = 2–10). On weekdays, most of the children were at school or childcare during the day (72.5%), and the rest stayed at their primary home address.

**Table 1:** Demographic characteristic of study participants (*n* = 40 parent–child pairs).

Characteristic^a^	*n* (%)
*Parents*	
Gender identity	
Female	38 (95)
Male	2 (5)
Age: mean (SD) = 36.6 (7.9) years	
20–35	19 (47.5)
36–50	19 (47.5)
>50	2 (5)
Race/Ethnicity^b^	
American Indian/Alaskan Native or Native Hawaiian/Other Pacific Islander	2 (5)
Asian	2 (5)
Black/African American	8 (20)
Hispanic/Latino	16 (40)
White	14 (35)
Prefer not to identify	1 (2.5)
Education	
High school diploma, GED, technical/trade school	8 (20)
Some college	8 (20)
College/graduate degree	24 (60)
Income	
0–$25,000	8 (20)
$25,000–$75,000	16 (40)
>$75,000	12 (30)
Prefer not to answer/don’t know	4 (10)
Work location	
At home^c^	19 (47.5)
Not at home	21 (52.5)
Smoking at home	
Yes	1 (2.5)
No	39 (97.5)
*Children*	
Gender identity	
Female	21 (52.5)
Male	19 (47.5)
Age: mean (SD) = 4.7 (2.1) years	
2–5	33 (82.5)
6–10	7 (17.5)
Race/Ethnicity^b,d^	
American Indian/Alaskan Native or Native Hawaiian/Other Pacific Islander	2 (5)
Asian	3 (7.5)
Black/African American	9 (22.5)
Hispanic/Latino	18 (45)
White	16 (40)
Prefer not to identify	2 (5)
Not reported	2 (5)
Daytime location	
Home	11 (27.5)
School/Childcare^e^	29 (72.5)

^a^Based on information collected during the first sampling round, unless otherwise noted.

^b^*N* greater than 40 because some participants reported multiple races/ethnicities.

^c^Includes stay-at-home parents.

^d^For some families, the child’s race/ethnicity was obtained during the second sampling round.

^e^Childcare includes care provided by a family member or a licensed childcare facility.

### Urinary 1-NP metabolites

**Fig. 1:**
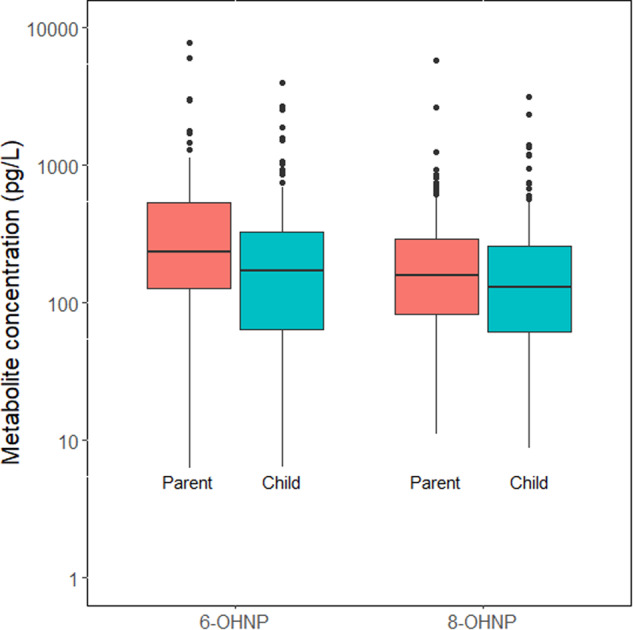
Box plot comparison of parent and child specific gravity-adjusted 1-NP metabolite concentrations (pg/L) in urine. The GM of 6-OHNP was significantly higher in parents than children (*p* = 0.00054).

Table 2 presents specific gravity-adjusted 1-NP metabolite concentrations and detection frequencies (DFs) for 6-OHNP and 8-OHNP in urine samples from parents and children (see SM Tables S2 and S3 for unadjusted and creatinine-adjusted values, respectively). Children’s urinary 6-OHNP concentrations were significantly lower (*p* = 0.005) compared with their parents (Fig. 1). Urinary 8-OHNP concentrations in children were also lower compared with parents, albeit not significantly (Fig. 1). For parents, 6-OHNP concentrations were higher than 8-OHNP concentrations (*p* < 0.001).

**Table 2:** Specific gravity-adjusted urinary 1-NP metabolite concentrations (pg/L) in parents and children^a^.

Group	Metabolite	*n*^b^	DF (%)	Minimum^c^	GM (95% CI)^d^	Median (IQR)^e^	95TH percentile	Maximum
*Parent*	6-OHNP	138	98	<LOD	240 (180, 310)	240 (130–540)	1500	7800
	8-OHNP	150	95	<LOD	150 (120, 190)	160 (82–290)	730	5800
*Child*	6-OHNP	155	94	<LOD	150 (110, 200)	170 (63–330)	1100	4000
	8-OHNP	168	95	<LOD	130 (100, 170)	130 (61–260)	740	3200

Summary statistics calculated using all reported values for each parent and child.

*IQR* Interquartile range (25th–75th percentiles).

^a^38 Parents and 40 children had valid 6-OHNP measurements. 40 parents and 40 children had valid 8-OHNP measurements.

^b^*n* = number of valid measurements.

^c^Urine LODs: 6-OHNP = 15.5 pg/L; 8-OHNP = 21.2 pg/L.

^d^GMs and CIs were calculated using a random-effects model that accounted for multiple samples from participants.

^e^The percentiles were based on all measurements without adjusting for multiple samples from an individual.

Parent and child 8-OHNP levels were weakly correlated (weighted Pearson *r* = 0.28, *p* < 0.001) but 6-OHNP levels were not significantly correlated (weighted Pearson *r* = 0.10, *p* = 0.24). Families with stay-at-home parents or parents who worked from home had stronger correlations between parent and child 1-NP metabolite levels (8-OHNP: weighted Pearson *r* = 0.41, *p* < 0.001, 19 families, 78 paired measurements; 6-OHNP: weighted Pearson *r* = 0.24, *p* = 0.04, 19 families, 72 paired measurements) than families in which the parent worked away from home (8-OHNP: weighted Pearson *r* = 0.20, *p* = 0.064, 21 families, 86 paired measurements; 6-OHNP: weighted Pearson *r* = -0.03, *p* = 0.83, 19 families, 66 paired measurements). We found that different definitions of daycare status (based on number of days in daycare or average time in daycare) resulted in different correlation results. We looked at the levels of 6-OHNP and 8-OHNP across all samples and found these were strongly correlated (weighted Pearson *r* = 0.84, *p* < 0.0001). A strong correlation between metabolites was also evident when measurements from parents and children were analyzed separately.

We examined GM concentrations of urinary 6-OHNP and 8-OHNP by income and race/ethnicity using mixed models that also accounted for seasonal effects (see SM Tables S4 through S7 for the specific gravity-adjusted GM concentrations for these categories). Metabolite concentrations were higher among parents in the highest annual family income category (>$75,000) compared with the other income categories (<$25,000 and $25,000–$75,000). After accounting for multiple comparisons using Tukey’s multiple comparisons test, significant differences were found between parents in the highest and lowest income groups for 6-OHNP and the highest and middle income groups for 8-OHNP. We did not observe any associations between urinary 1-NP metabolite concentrations and race/ethnicity.

**Fig. 2:**
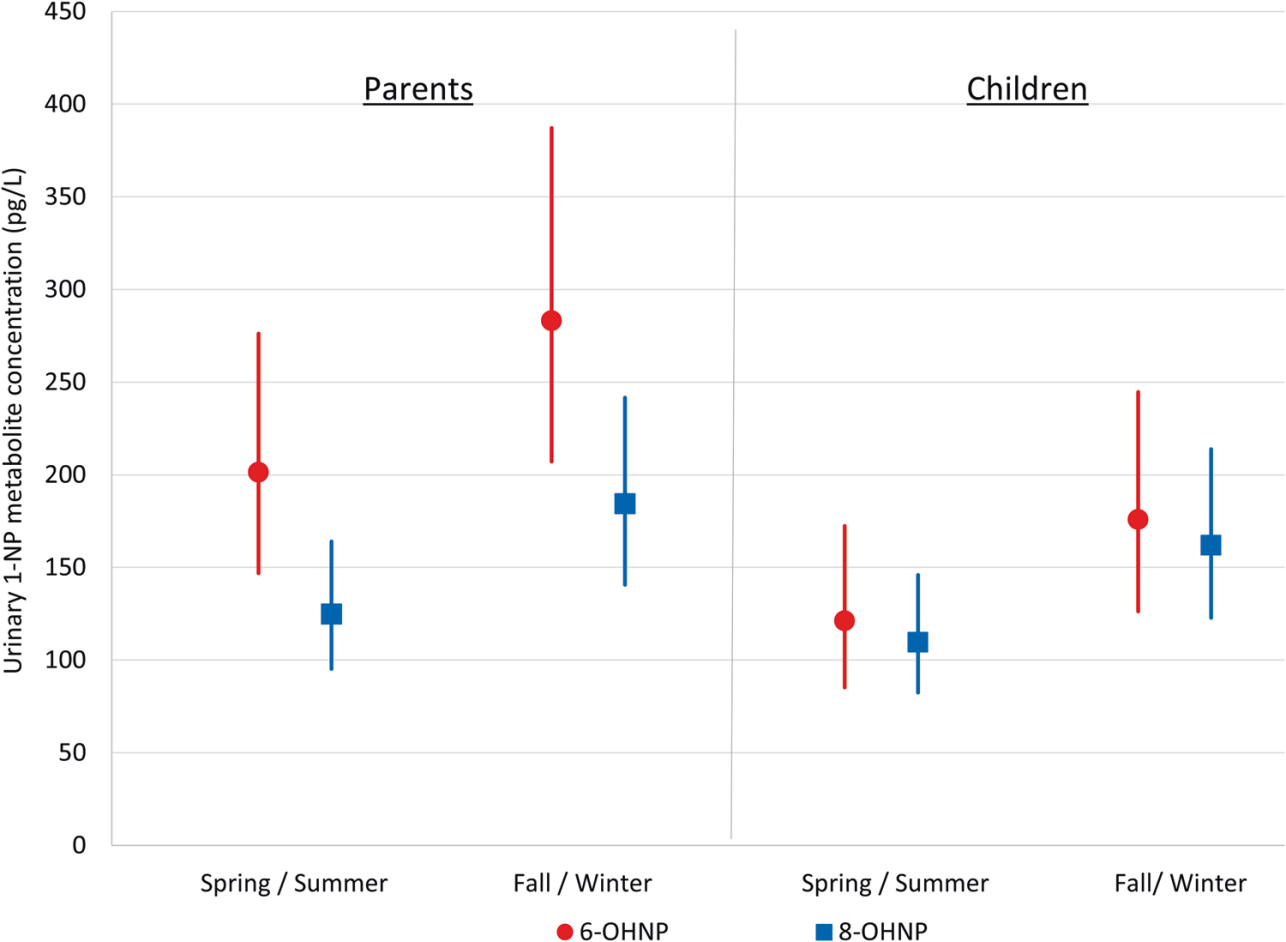
GM concentrations of urinary 1-NP metabolite concentrations (adjusted for specific gravity) in parents and children by seasonal period. Error bars indicate 95% CIs. The GMs were significantly greater in fall/winter than in spring/summer for 8-OHNP (*p* = 0.017 for children and *p* < 0.01 for parents) and nearly so for 6-OHNP (*p* = 0.070 for children and *p* = 0.053 for parents).

Figure 2 plots the estimated GMs of the specific gravity-adjusted urinary 6-OHNP and 8-OHNP concentrations for parents and children by seasonal period (also see SM Tables S4 through S7). In parents and children, the GMs of the urinary metabolites were higher in fall/winter than in spring/summer. The mixed model used to estimate the GMs indicated significant differences by seasonal period in 8-OHNP concentrations for both children and parents (*p* = 0.017 for children and *p* < 0.01 for parents) and borderline significant differences in 6-OHNP concentrations for both children and parents (*p* = 0.070 for children and *p* = 0.053 for parents).

Table 3 shows the within- and between-subject variability in 1-NP metabolites from daily urine samples collected over four days during each of two sampling rounds (*n* = 15 parent–child pairs). ICCs were 0.39 and 0.43 for 6-OHNP and 8-OHNP concentrations in parents, and 0.41 and 0.38 in children. Consistent with the relatively low ICC values, within-subject variance was higher than between-subject variance (57–62% versus 38–43% of total variance) (Table 3). These results indicate generally weak correlation and high temporal variability for repeated measures of 1-NP metabolite concentrations over short time frames (days) and are consistent with a half-life <24 h in humans [15].

**Table 3:** Variance apportionment of log-transformed urinary 1-NP metabolite concentrations within sampling round for daily spot urine samples collected over 4 days from 15 parent–child pairs.

Group	6-OHNP				8-OHNP			
	*n*^a^	Variance	Percent of total variance	ICC	*n*^a^	Variance	Percent of total variance	ICC
*Parent*	95				102			
Between		0.10	39%	0.39		0.09	43%	0.43
Within		0.16	61%			0.12	57%	
*Child*	106				117			
Between		0.13	41%	0.41		0.08	38%	0.38
Within		0.19	59%			0.14	62%	

Variance computed using linear mixed-effects models, with participant set as a random effect variable and sampling round (1st or 2nd) set as a fixed effect variable.

*ICC* intraclass correlation coefficient.

^a^*n* = number of valid measurements.

### 1-NP in household dust and indoor air

Table 4 presents the household dust and indoor air concentrations of 1-NP. The GM (95% CI) concentration of 1-NP in dust was 380 (200, 730) pg/g with a 97% DF. One dust sample, collected from a home situated near the intersection of multiple freeways, had an extremely high 1-NP concentration (910,000 pg/g; approximately 200-fold more than the next highest sample). Extensive quality assurance review of this sample analysis did not indicate any laboratory errors. The GM (95% CI) concentration of 1-NP in indoor air was 0.41 (0.36, 0.45) pg/m^3^ with a DF of 74%. The lower DF in indoor air could be related to the low flow rate of the ABCD pumps used in EBDEP, resulting in a lower total air volume sampled than in prior studies of 1-NP [30]. Log-transformed indoor air and dust 1-NP concentrations from sampling round 1 were moderately correlated (Pearson *r* = 0.54, *p* < 0.01 when the dust sample with the extremely high 1-NP concentration was included; *r* = 0.45, *p* < 0.05 when that sample was excluded).

**Table 4:** 1-Nitropyrene concentrations in household dust (pg/g) and indoor air (pg/m^3^).

Median	*n*^a^	DF (%)	Minimum^b^	GM (95% CI)	Median (IQR)	95th percentile	Maximum
Dust	36^c^	97	<LOD	380 (200, 730)	340 (190–620)	4500	910,000^d^
Air	74	74	<LOD	0.41 (0.36, 0.45)^**e**^	0.43 (<LOD − 0.54)^f^	0.87^f^	1.2

*IQR* interquartile range (25th–75th percentiles).

^a^*n* *=* number of valid measurements.

^b^Dust LOD = 15 pg/g; air LOD = 0.3 pg/m^3^.

^c^Thirty-six dust samples were collected from 33 homes. For homes with multiple dust samples, the average 1-NP concentration was used for calculating summary statistics.

^d^This measurement was validated; see the “Results” section for more details.

^e^The GM and 95% CI for air measurements were calculated using a random-effects model to account for multiple measurements from each home.

^f^The percentiles were based on all measurements without adjusting for multiple samples from a home.

Consistent with the findings reported above for the urinary 1-NP metabolites, 1-NP concentrations in indoor air were higher in fall/ winter (GM [95% CI] = 0.47 [0.41, 0.55]) compared with spring/summer (0.35 [0.30, 0.41]) (*p* < 0.01).

We examined correlations between 1-NP in dust and indoor air and 1-NP metabolites in urine samples collected during sampling round 1. The extremely high dust observation was excluded from this analysis. Urinary 8-OHNP levels in children were weakly correlated with 1-NP in dust (weighted Pearson *r* = 0.22, *p* = 0.02) and indoor air (weighted Pearson *r* = 0.22, *p* < 0.01). There were no other statistically significant correlations between 1-NP in dust or indoor air and urinary metabolites.

### Comparisons with CalEnviroScreen diesel PM scores

We compared our results with the CalEnviroScreen 3.0 diesel PM scores used to target areas for EBDEP recruitment. Six families were in the lower exposure areas (scores at or below the 50th percentile). GMs for 1-NP in house dust were higher for census tracts with diesel PM scores above the 90th percentile than for those below (*t*-test *p* = 0.027 with the extreme dust value excluded). GMs for 1-NP in indoor air were higher for census tracts with diesel PM scores above the 90th percentile (mixed model *p* = 0.017). Mixed models did not indicate a significant difference in 1-NP metabolites for parents or children between these two census tract groups, with and without seasonal effects in the models.

## Discussion

EBDEP is the first study to evaluate DE exposures in families with children as young as 2 years old by combining 1-NP biomonitoring with measurements in household dust and indoor air. Metabolites of 1-NP were present in the urine of almost all participating parents and children (DFs > 90%), indicating widespread DE exposures in these populations. In both children and parents, we observed a wide range of 1-NP metabolite levels, with the 95th percentiles several times the GMs, and maximum values more than an order of magnitude higher, indicating that some participants had much higher exposures than other participants. Specific gravity-adjusted metabolite concentrations were generally higher in parents compared with children. We will examine possible explanations for this difference in future analyses.

The differences we observed in urinary 1-NP metabolite levels and 1-NP levels in indoor air by seasonal period suggest that higher DE exposures occur during fall/winter. This finding was expected based on prior studies that have demonstrated increased air pollution linked to periodic temperature inversions, which can trap pollutants in residential areas along major transportation corridors in the San Francisco East Bay [34]. Our observations of differences in 1-NP levels by seasonal period are limited by EBDEP’s small sample size.

The small sample size also limited our ability to examine associations of 1-NP metabolite levels with demographic factors. Parents’ urinary 1-NP metabolite levels tended to be higher in families with higher incomes. We did not find significant associations between urinary 1-NP metabolite concentrations and race/ethnicity.

1-NP metabolite levels in EBDEP parents were higher than levels measured in the California Regional Exposure Study conducted in Los Angeles County (CARE-LA), a Biomonitoring California study of LA County adults. The EBDEP GMs of 240 pg/L and 150 pg/L, for 6-OHNP and 8-OHNP respectively, were about two times higher than the CARE-LA GMs of 110 and 88 pg/L [35]. However, CARE-LA was designed to examine the general population, so recruited participants from all over LA County and not specifically in areas with high diesel exhaust emissions. In addition, CARE-LA samples were collected primarily in the spring (between February and May) while EBDEP samples were collected over more seasons. These factors could account for the lower levels found in CARE-LA.

In a study of adults crossing the US/Mexico border, Galaviz et al. [13] reported GM levels of 8-OHNP that were less than half of what we observed in EBDEP parents. Levels of 1-NP metabolites that have been measured in occupationally exposed populations were higher than in EBDEP parents. For example, in Chinese taxi drivers, the GMs were 3 times higher for 6-OHNP and 7 times higher for 8-OHNP [15]. In a study of Peruvian traffic workers, the GMs were more than an order of magnitude higher than those measured in EBDEP parents [22].

EBDEP results showed high within- versus between-subject variability of urinary 1-NP metabolites, indicating that multiple urine samples would be needed over weeks or months to characterize exposures over long time periods. This finding is consistent with observations for other chemicals with short biological half-lives [36].

EBDEP is one of the first studies to report concentrations of 1-NP in household dust. The high DF (97%) we observed indicated widespread occurrence of 1-NP in participants’ homes. Besis et al. measured 1-NP in dust samples collected in 2017 from 21 houses in Greece [37]. They found a median of 838 pg/g, roughly double the EBDEP median. The maximum 1-NP value Besis et al. observed was 8670 pg/g, which was well below the extreme value in EBDEP (910,000 pg/g).

Young children often spend time indoors on floors and have frequent hand-to-mouth behaviors [38, 39], suggesting they are more likely than adults to be exposed to 1-NP through non-dietary ingestion and dermal absorption in addition to inhalation. We found that urinary 8-OHNP was correlated with 1-NP in dust for children, but not for adults.

The median 1-NP concentration (0.42 pg/m^3^) in indoor air of EBDEP homes was similar to the median *outdoor* level (0.49 pg/m^3^) reported in Seattle, Washington in 2012 [40]. In contrast, a 2014 study along the San Ysidro US–Mexico border [41] reported a median outdoor air level that was three times higher (1.3 pg/m^3^) than what we observed in indoor air in EBDEP.

High CalEnviroScreen diesel PM scores were related to higher levels of 1-NP in indoor air and house dust samples. However, we did not detect a relationship between the diesel PM scores and urinary 1-NP metabolite concentrations. One possible explanation for this difference is that the air and dust samples were collected from stationary homes, while the urine samples were collected from participants who often moved through different locations. A further consideration is that the diesel PM scores in CalEnviroScreen 3.0 were estimated for census tracts based on modeling emissions over a 4 km^2^ statewide grid, while the EBDEP air and dust measurements were localized to participant homes. Prior studies have shown that air pollution concentrations can vary greatly over small distances in urban areas [9, 42]. Hyperlocal air monitoring and biomonitoring are needed to define exposures more specifically, augmenting information from ambient air monitoring networks and emissions inventories. Hyperlocal measurements can also better map inequities between communities.

In future analyses, we will examine potential determinants of 1-NP metabolite concentrations in urine and 1-NP levels in household dust and indoor air, including traffic, rail, and maritime sources proximate to the locations where participants spent time (i.e., residence, work location, childcare facility, or school). Given the high short-term variability of 1-NP metabolite levels in urine, we will examine factors that could modify associations between DE sources and exposures, such as meteorological events that change air quality over short time periods (e.g., changes in wind direction or precipitation events that wash out pollutants).

EBDEP was the first step in our efforts to use biomonitoring for evaluating air pollution exposures in heavily burdened communities in California. Biomonitoring studies, coupled with ongoing community air monitoring, will improve understanding of hyperlocal air pollution impacts and support the evaluation of effective mitigation strategies.

## Supplementary information

Supplementary Material

## Data Availability

De-identified data that support the findings of this study are available upon request. Certain study data cannot be made publicly available due to IRB restrictions prohibiting the sharing of information that could compromise research participant privacy upon which consent was contingent.
