# Supplementary material for: The East Bay Diesel Exposure Project: a biomonitoring study of parents and their children in heavily impacted communities
Source: J Expo Sci Environ Epidemiol. 2023 Dec 15;34(5):827–35. doi: 10.1038/s41370-023-00622-1 (PMC11446841; doi:10.1038/s41370-023-00622-1)

Supplementary Material

The East Bay Diesel Exposure Project: A biomonitoring study of parents and their children in heavily impacted communities

Daniel Sultana[1], Duyen Kauffman[1,2], Rosemary Castorina[3], Michael H. Paulsen[4], Russell Bartlett[1,2], Kelsey Ranjbar[1], Robert B. Gunier[3], Victor Aguirre[1], Marina Rowen[3], Natalia Garban[1], Josephine DeGuzman[5], Jianwen She[5], Regan Patterson[1,6], Christopher D. Simpson[4], Asa Bradman[3,7*], Sara Hoover[1]

Authors' affiliations
[1]Office of Environmental Health Hazard Assessment (OEHHA), California Environmental Protection Agency, Oakland, CA
[2]Environmental Health Investigations Branch, California Department of Public Health, Richmond, CA.
[3]Center for Environmental Research and Community Health (CERCH), School of Public Health, University of California, Berkeley, CA
[4]Department of Environmental and Occupational Health Sciences, School of Public Health, University of Washington, Seattle, WA
[5]Environmental Health Laboratory, California Department of Public Health, Richmond, CA
[6]Department of Civil and Environmental Engineering, University of California, Berkeley, CA
[7]University of California, Merced, Department of Public Health, Merced, CA
11 pages, 7 tables, 2 figures

***Corresponding author:**

Asa Bradman, PhD, MS
University of California, Merced
Department of Public Health
5200 North Lake Rd
Merced, CA 95343 USA
PH: (209) 228-4400
abradman@ucmerced.edu

Table of Contents

Table S1. Sample and data collection scheme (n = 40 parent-child pairs) 3

Table S2. Unadjusted urinary 1-NP metabolite concentrations (pg/L) in parents and children 4

Table S3. Creatinine-adjusted urinary 1-NP metabolite concentrations (pg/g-creatinine) in parents and children 5

Table S4. Specific gravity-adjusted urinary 6-OHNP concentrations (pg/L) in parents by ethnicity, family income, and seasonal period 6

Table S5. Specific gravity-adjusted urinary 8-OHNP concentrations (pg/L) in parents by ethnicity, family income, and seasonal period 7

Table S6. Specific gravity-adjusted urinary 6-OHNP concentrations (pg/L) in children by ethnicity, family income, and seasonal period 8

Table S7. Specific gravity-adjusted urinary 8-OHNP concentrations (pg/L) in children by ethnicity, family income, and seasonal period 9

Figure S1. General locations of EBDEP study participants' residences......................................10

Figure S2. CalEnviroScreen diesel PM results in the East Bay Area 11

Table S1. Sample and data collection scheme (n = 40 parent-child pairs)

	Initial exposure questionnaire and home walk-through	Dust sample[a]	Single urine sample (n = 25)	Daily urine samples[b] (n = 15)	Air sample	Follow-up exposure questionnaire
Day 1	✓	✓		✓	Detector deployed	
Day 2				✓	↓	
Day 3				✓		
Day 4			✓	✓	Detector retrieved and filter collected	✓

[a]Dust samples were collected only in the first sampling round.

[b]Daily urine samples were collected by a subset of participants (n = 15 parent-child pairs). All urine samples were retrieved by study staff on day 4 of the sampling round.

Table S2. Unadjusted urinary 1-NP metabolite concentrations (pg/L) in parents and children[a]

Group	Metabolite	Minimum[b]	GM (95% CI)[c]	Median (IQR)[d]	95th percentile	Maximum
Parent	6-OHNP	< LOD	190 (150, 240)	210 (100, 360)	880	7,400
	8-OHNP	< LOD	120 (96, 150)	130 (67, 230)	630	4,400
Child	6-OHNP	< LOD	160 (120, 210)	180 (76, 350)	1,300	3,500
	8-OHNP	< LOD	150 (120, 190)	155 (80, 295)	840	4,100

Notes:
Summary statistics calculated using all reported values for each parent and child.
IQR = Interquartile range (25th - 75th percentiles)

[a]38 parents and 40 children had valid 6-OHNP measurements. 40 parents and 40 children had valid 8-OHNP measurements.
[b]Urine LODs: 6-OHNP = 15.5 pg/L; 8-OHNP = 21.2 pg/L
[c]GMs and CIs were calculated using a random effects model that accounted for multiple samples from participants.
[d]The percentiles were based on all measurements without adjusting for multiple samples from an individual.

Table S3. Creatinine-adjusted urinary 1-NP metabolite concentrations (pg/g-creatinine) in parents and children[a]

Group	Metabolite	Minimum[b]	GM (95% CI)[c]	Median (IQR)[d]	95th percentile	Maximum
Parent	6-OHNP	< LOD	210 (160, 280)	230 (100, 530)	1,600	9,400
	8-OHNP	< LOD	130 (100, 170)	140 (72, 290)	720	6,900
Child	6-OHNP	< LOD	250 (180, 340)	270 (95, 610)	1,600	8,100
	8-OHNP	< LOD	230 (170, 300)	210 (100, 510)	1,300	6,500

Notes:
Summary statistics calculated using all reported values for each parent and child.
IQR = Interquartile range (25th - 75th percentiles)

[a]38 parents and 40 children had valid 6-OHNP measurements. 40 parents and 40 children had valid 8-OHNP measurements.
[b]Urine LODs: 6-OHNP = 15.5 pg/L; 8-OHNP = 21.2 pg/L
[c]GMs and CIs were calculated using a random effects model that accounted for multiple samples from participants.
[d]The percentiles were based on all measurements without adjusting for multiple samples from an individual.

Table S4. Specific gravity-adjusted urinary 6-OHNP concentrations (pg/L) in parents by ethnicity, family income, and seasonal period

**Category**		**Number of valid measurements**	**GM (95th CI)**
**Race/ethnicity– parent**[a]	Black/African American	27	200 (110, 360)
	Hispanic/Latino	44	220 (140, 340)
	White	46	270 (180, 420)
	Other or multi-racial[b]	10	170 (83, 350)
**Family income**[a]	<$25,000	24	160 (90,270)
	$25,000-$75,000	66	200 (140, 280)
	>$75,000	36	370 (240, 570)
**Seasonal period**	Spring/summer	62	200 (150, 280)
	Fall/winter	76	280 (210, 390)

[a]The models used to estimate the geometric means for race/ethnicity and family income included a seasonal effect.
[b]Category includes Asian; American Indian/Alaskan Native or Native Hawaiian/Other Pacific Islander; and individuals who selected more than one ethnicity. Individuals who preferred not to identify ethnicity were not included in this analysis.

Table S5. Specific gravity-adjusted urinary 8-OHNP concentrations (pg/L) in parents by ethnicity, family income, and seasonal period.

**Category**		**Number of valid measurements**	**GM (95th CI)**
**Race/ethnicity - parent**[a]	Black/African American	28	180 (110, 300)
	Hispanic/Latino	45	110 (79, 160)
	White	54	160 (120, 230)
	Other or multi-racial[b]	12	140 (75, 240)
**Family income**[a]	<$25,000	26	120 (73, 190)
	$25,000-$75,000	69	120 (88, 160)
	>$75,000	41	230 (160, 320)
**Seasonal period**	Spring/summer	67	120 (95, 160)
	Fall/winter	83	180 (140, 240)

[a]The models used to estimate the geometric means for race/ethnicity and family income included a seasonal effect.
[b]Category includes Asian; American Indian/Alaskan Native or Native Hawaiian/Other Pacific Islander; and individuals who selected more than one ethnicity. Individuals who preferred not to identify ethnicity were not included in this analysis.

Table S6. Specific gravity-adjusted urinary 6-OHNP concentrations (pg/L) in children by ethnicity, family income, and seasonal period.

**Category**		**Number of valid measurements**	**GM (95th CI)**
**Race/ethnicity – child**[a]	Black/African American	17	84 (41, 170)
	Hispanic/Latino	36	150 (93, 240)
	White	49	130 (80, 200)
	Other or multi-racial[b]	38	170 (110, 260)
**Family income**[a]	<$25,000	26	110 (58, 210)
	$25,000-$75,000	69	150 (100, 220)
	>$75,000	44	180 (110, 290)
**Seasonal period**	Spring/summer	62	120 (85, 170)
	Fall/winter	93	180 (130, 240)

[a]The models used to estimate the geometric means for race/ethnicity and family income included a seasonal effect.
[b]Category includes Asian; American Indian/Alaskan Native or Native Hawaiian/Other Pacific Islander; and individuals who selected more than one ethnicity. Individuals who preferred not to identify ethnicity were not included in this analysis.

Table S7. Specific gravity-adjusted urinary 8-OHNP concentrations (pg/L) in children by ethnicity, family income, and seasonal period.

**Category**		**Number of valid measurements**	**GM (95th CI)**
**Race/ethnicity – child**[a]	Black/African American	17	85 (45, 160)
	Hispanic/Latino	39	130 (85, 190)
	White	58	120 (78, 170)
	Other or multi-racial[b]	38	160 (110, 240)
**Family income**[a]	<$25,000	27	98 (58, 170)
	$25,000-$75,000	73	140 (99, 200)
	>$75,000	50	160 (110, 230)
**Seasonal period**	Spring/summer	72	110 (82, 150)
	Fall/winter	96	160 (120, 210)

[a]The models used to estimate the geometric means for race/ethnicity and family income included a seasonal effect.
[b]Category includes Asian; American Indian/Alaskan Native or Native Hawaiian/Other Pacific Islander; and individuals who selected more than one ethnicity. Individuals who preferred not to identify ethnicity were not included in this analysis.

Figure S1. General locations of EBDEP study participants' residences

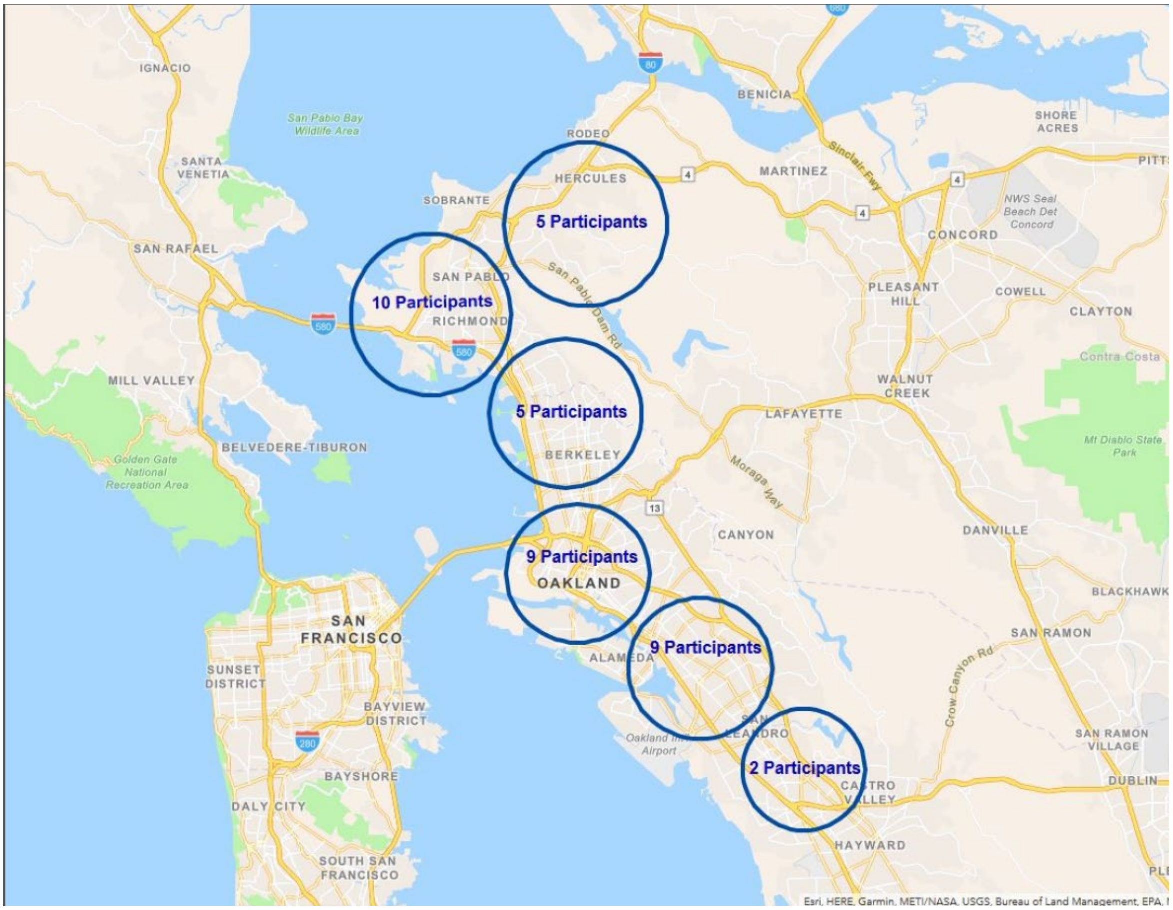

Figure S2. CalEnviroScreen diesel PM results in the East Bay Area

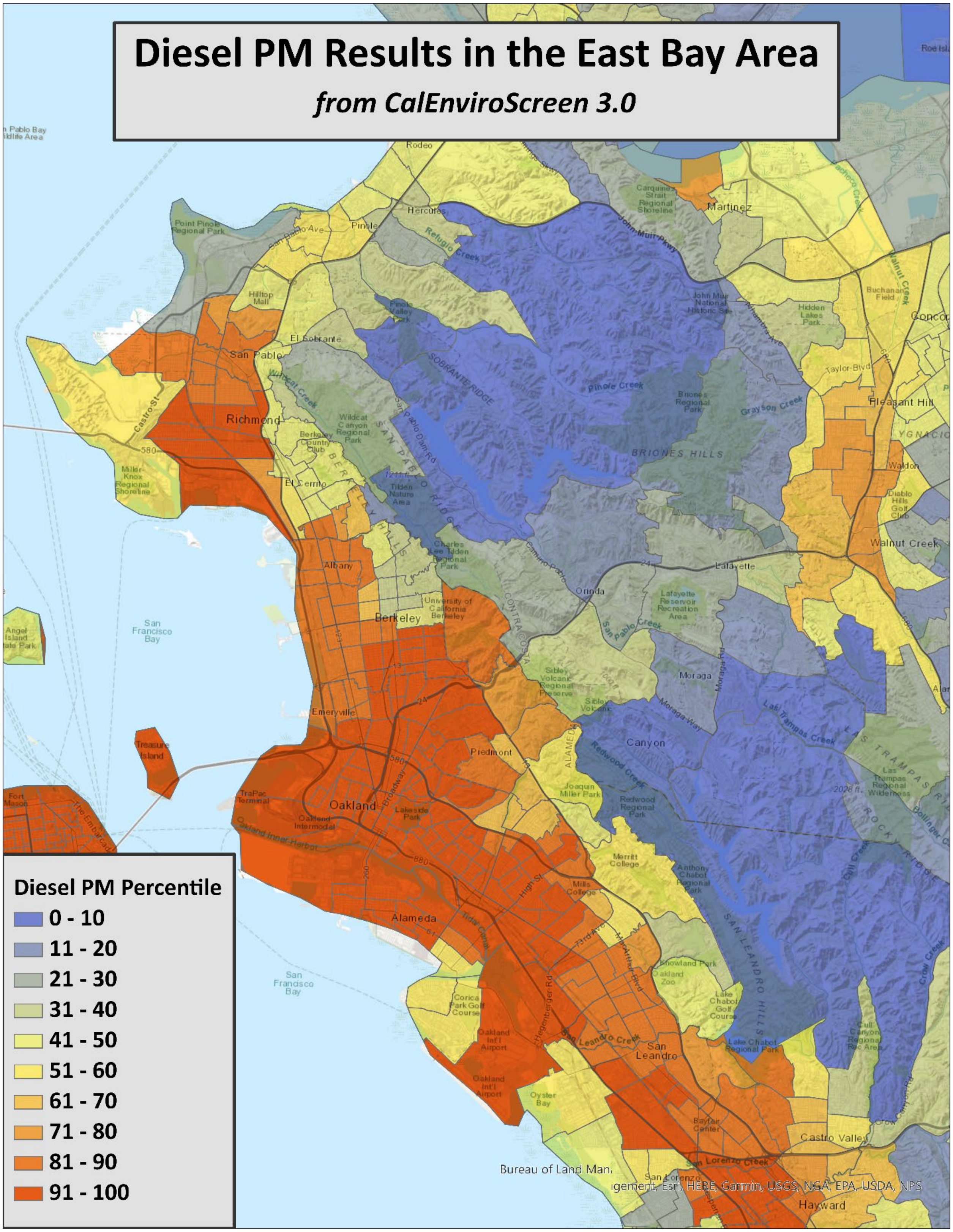

Supplement: Supplementary file 1 — Supplementary Material [file 41370_2023_622_MOESM1_ESM.pdf]
